# The impact of reintroduced Eurasian beaver (*Castor fiber*) dams on the upstream movement of brown trout (*Salmo trutta*) in upland areas of Great Britain

**DOI:** 10.1371/journal.pone.0313648

**Published:** 2025-02-13

**Authors:** Robert J. Needham, Richard W. Zabel, Dylan Roberts, Paul S. Kemp

**Affiliations:** 1 International Centre for Ecohydraulic Research (ICER), Boldrewood Innovation Campus, University of Southampton, Southampton, United Kingdom; 2 National Marine Fisheries Service, Northwest Fisheries Science Center, Seattle, Washington, United States of America; 3 Game and Wildlife Conservation Trust, Fordingbridge, Hampshire, United Kingdom; KTH Royal Institute of Technology: Kungliga Tekniska Hogskolan, SWEDEN

## Abstract

The return of Eurasian beaver (*Castor fiber*) to large areas of Europe represents a conservation success with the current population estimated to be around 1.2 million individuals. Their reintroduction to many areas, including Great Britain, has in some cases been controversial. Despite numerous documented benefits to biodiversity, concerns relate to localised flooding, adverse impacts on land use and engineered structures (e.g. culvert blockage), disease transfer, and the influence of beaver habitat modifications on fisheries, particularly in relation to salmonids. This study investigated the impacts of a series of four beaver dams on the upstream movement of brown trout during the spawning period (October—December) at a field site in Scotland. The study site comprised two streams entering a common loch, one modified by a series of four beaver dams, the other remaining unaltered during the Study Period. Trout were captured using electric fishing, fyke nets and rod and line and were tagged with Passive Integrated Transponders (PIT) before release. PIT telemetry antennas were installed below and above each dam to establish successful passage of trout during the monitoring period that included trout spawning movements in 2015 (high flows) and 2016 (low flow). There was a distinct difference in passage success between years, with high flows (using prior rainfall as a proxy measure) and larger fish size being important positive predictors of upstream passage success. A combination of environmental (prior rainfall and water temperature) and biotic (fish size) factors influenced passage success with high flows being a significant covariate at all four dams in two models used to define trout passage dynamics (Weibull and exponential base models), providing the best explanatory variable for fish passage at two of the four dams. Survival analysis and associated modelling indicated that migratory delay was inversely related to previous passage success, whilst motivation was also a determinant of success, with greatest passage in highly motivated trout. Our findings indicate that given the right environmental and biotic factors, brown trout are adept at passing beaver dams, although under certain conditions, beaver dams can impede the movement of brown trout and the magnitude of impact is influenced by these factors. In particular, the barrier effects of beaver dams are exacerbated under low flow conditions, and this may become a greater challenge in the future due to shifting climatic conditions if periods of warmer and drier weather persist and coincide with peak migratory movements of fish.

## Introduction

The return of the Eurasian beaver (*Castor fiber*) to large areas of Europe is a conservation success. At the beginning of the 20^th^ Century, the beaver had been so intensively hunted that only approximately 1,200 remained, and these were restricted to a few relic populations across Europe [1]. Since then, the population has increased by around three orders of magnitude to approximately 1.2 million animals through natural range expansion and a series of reintroductions [2]. Compared to elsewhere in Europe, however, the return of beaver to Britain has been a relatively recent development. A licensed trial reintroduction in Knapdale Forest, Argyll, Scotland, combined with a series of accidental escapes and unauthorised releases (in England: River Otter and Tamar catchments, Devon; Rivers Avon, Wiltshire; Frome catchments, Somerset; River Stour, Kent; in Scotland: River Tay catchment, Perthshire) has led to six self-sustaining and expanding populations of wild living beaver being established. Due to the increase in abundance and range, beavers are now afforded protection as a ‘European Protected Species’ in Scotland and England under retained EU law (Annex IV [a] EC Habitats and Species Directive [ECC 92/43]).

Beaver reintroductions throughout Europe have been controversial with multiple stakeholders articulating differing perspectives. Concerns include localised flooding due to dam construction and failure following heavy rains; negative impacts on land use, such as agriculture and forestry through burrowing, canal construction, damming of smaller watercourses; crop foraging; and felling of commercially important timber [3]. Furthermore, beaver dams and associated accumulation of woody material may disrupt the functioning of engineered structures [4], e.g. by blocking culverts, weirs, sluices and fish passes, with burrowing activities possibly compromising flood defences [3], resulting in sub-optimal operation and occasional costly failure in North America [5] and Scotland [6]. Concerns regarding disease transmission have also been raised by regulatory bodies (e.g., DEFRA, 2012; IUCN/SSC, 2014) as Eurasian beavers are potential hosts for a range of infectious diseases (e.g. Leptospirosis) and parasites (e.g., *Cryptosporidium parvum* and *Echinococcus multilocularis*) [7, 8]. However, it is the impact of beaver habitat modification on fish and fisheries that, for some at least, is of greatest concern [9].

From the perspective of stream-dwelling fish, considerations of which are typically directed towards the economically important salmonids [10–13], concerns relate to the potential for beaver dams to degrade and fragment fluvial habitats [14–16]. More specifically, beaver dams may reduce the availability of suitable salmonid spawning habitat if traditional breeding grounds are impounded [13]. By regulating flow and reducing velocities downstream, beaver dams can cause the deposition of fine sediment that infiltrates the gravels in which salmonids spawn [12, 17]. This can increase egg mortality by limiting oxygen replenishment and reducing the removal of metabolic waste products [18]. Furthermore, beaver dams may impede fish movements, particularly in tributary streams, thus preventing them reaching critical habitats, e.g. for spawning or rearing [14].

Observations on the extent to which beaver dams hinder the upstream movements of salmonids are variable and contradictory. Some studies provide examples of extensive delays of Atlantic salmon, *Salmo salar* at North American beaver, *Castor canadensis*, dams [12, 13], whereas others report limited impacts on Atlantic salmon and Brown trout, *Salmo trutta* at Eurasian beaver dams [19, 20]. The explanations for such variability are likely to be context dependent, with magnitude of the impact influenced by a number of secondary factors that may be either environmental (e.g. dam density when in a complex [10], dam age and condition [10, 11] and probability of dam breach and hydrological linkages [10]) or biotic (for anthropogenic structures these include motivation [21], life stages [22], personality [23], and life history strategy [24].) From the environmental perspective, however, it is the interaction with river flow that is likely to be the primary factor governing the impact of beaver dams on fish movements [11], with greatest effects observed during periods in which low flows coincide with fish migration [12, 13].

Over the next few decades, a shifting climate is expected to result in increasingly drier UK summers [25] extending into the Autumn [26], with regions such as Scotland experiencing greater frequency and intensity of drought [27]. Under such scenarios, the impact of barriers to migration under low flow scenarios may pose an ever-greater challenge to fish population viability. This study investigated the impacts of a series of four beaver dams on the upstream movement of brown trout during the spawning period (October—December) at a field site in Scotland. Using continuous fine resolution PIT telemetry during two spawning seasons (2015 and 2016) we provided: (1) a coarse resolution description of passage efficiency; (2) fine-scale quantification of delay at each dam accounting for environmental (temperature and rainfall) and biotic (fish size) factors; and (3) the influence of individual motivation, a frequently ignored but important fish passage metric [28]. We made three predictions: (P1) passage efficiency and (P2) delay would be respectively positively and negatively related to flow and (P3) swimming speed between dams will be greatest for not only the larger fish, but also the most motivated. This study quantified the impacts of beaver dams on the movement of trout and provides useful insight to inform strategies to manage the influence of reintroduced beavers on fish populations within the UK context.

## Methods

### Study site

**Fig 1:**
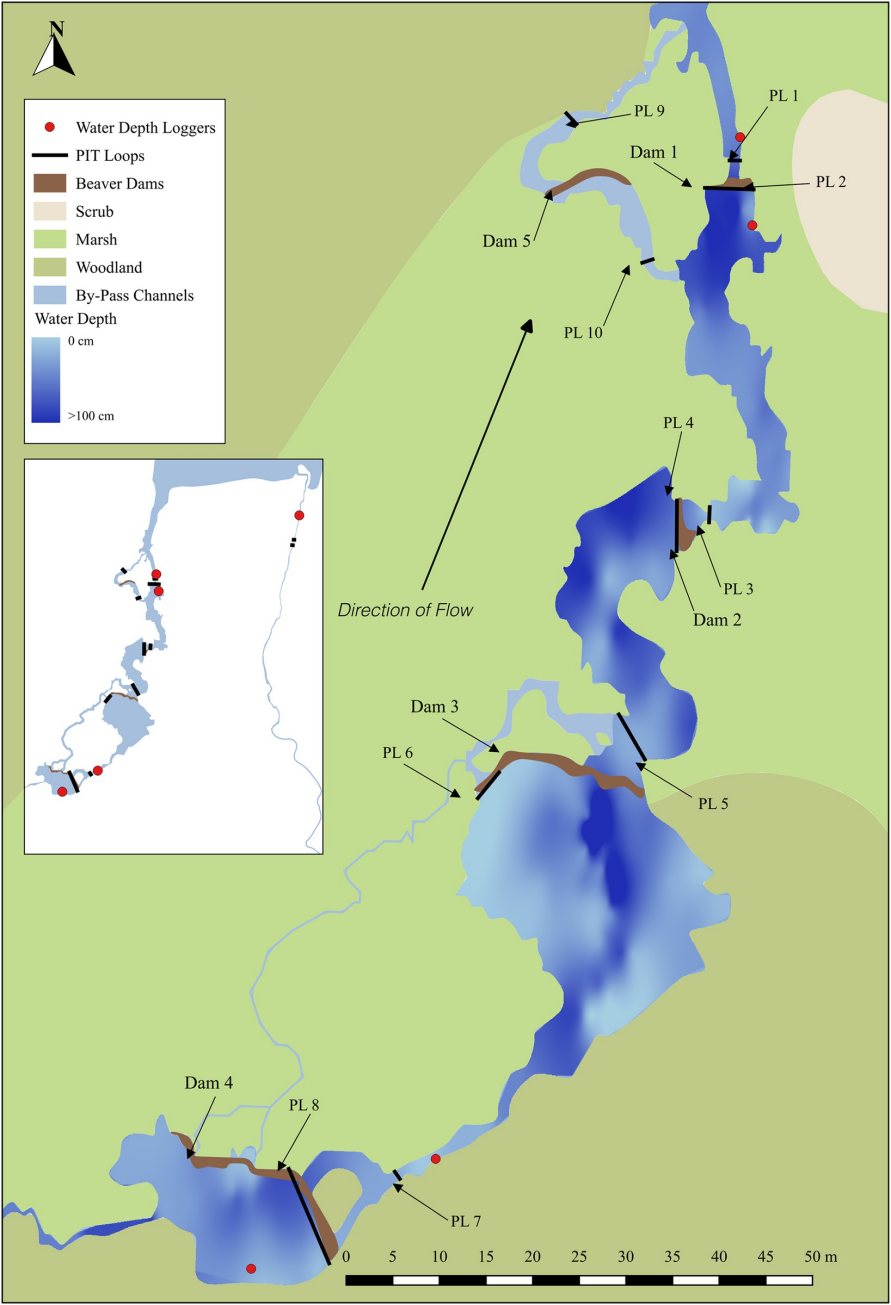
Map of study site in Northern Scotland. Study site in which the movements of brown trout (*Salmo trutta*) in response to fluvial landscape modification by Eurasian beaver (*Castor fiber*) was investigated. The map illustrates the modified stream post beaver modification and the surrounding landscape and habitat types. The inset map illustrates an overview of the site with the loch in the north and control stream to the east of the modified stream. The position of beaver dams, passive integrated transponder (PIT) loops (to monitor fish movement), and water data loggers (depth and temperature) are indicated.

The Allt Coire an t- Seilich (beaver modified) and Allt a’ Choilich (control) are two first order streams that flow in a northeast direction before entering an impounded loch, known locally as Loch Grant (17,644 m^2^; NH5452940584; ca 160 m.a.s.l; Fig 1). The loch outflow continues as the Allt a’ Choilich, flowing northeast for 2 km before joining the Moniack Burn, which discharges directly into the Beauly Firth, Inverness-shire, Scotland.

Both modified and control streams exhibited similar physical, geomorphological and hydrological characteristics prior to beaver modification (Chris Swift [landowner], personal communication, 2014). The modified stream was impounded in four locations by beaver dams, constructed and maintained since release in 2007, (see Table 1 for dimensions) to create four ‘modified’ reaches (mean length: 51.75 m) with an additional dam (Dam 5, Table 1 and Fig 1) constructed to the west of Dam 1 in 2016. The control site was similarly divided into four ‘control’ reaches determined by riparian vegetation and accessibility (mean length: 34.5 m) and remained unmodified by beavers during the study.

**Table 1:** Dimensions of dams constructed by Eurasian beavers released in 2008 on the Allt Coire an t- Seilich Burn in Inverness-shire, Scotland, and installed Passive Integrated Transponder (PIT) loop (PIT Loops (PL)) dimensions, detection range (distance from loop at first detection) and detection efficiency (percentage of 30 tags detected when manually passed through the loop). Measurements: Dam Crest Width—distance across the top of structure; Dam Height—distance from the water surface to top of dam; Water Depth Below Dam—distance from stream bed to stream surface. Dam 2 was original installed as a Beaver Dam Analogue (BDA) with fence posts driven into the stream bed in 2007 to encourage recently released beavers to build, this has been added to and now maintained as a beaver dam. *In spring 2016 an additional dam (Dam 5) was constructed on the Dam 1 by-pass channel.

	Dam Crest Width (m)	Dam Height (m)	Water Depth Below Dam (m)	By-Pass Channel	PIT Loop (PL)			Detection Range (m)		Detection Efficiency (%)	
					(PL)	Width (m)	Height (m)	12mm	23mm	12mm	23mm
Dam 1	5.1	0.56	0.47	*Yes	1	2.00	0.80	0.28	0.87	100	100
					2	5.85	0.45	0.05	0.32	100	100
Dam 2	5.8	0.57	0.26	No	3	3.00	0.80	0.31	1.21	100	100
					4	5.85	0.43	0.12	0.60	100	100
Dam 3	19.3	0.55	0.13	Yes	5	3.35	0.90	0.10	0.64	97	100
					6	2.60	0.50	0.22	0.67	100	100
Dam 4	24	0.97	0.19	No	7	1.35	0.55	0.25	0.84	100	100
					8	8.00	0.30	0.17	0.50	100	100
*Dam 5	10.1	0.54	0.13	No	9	0.65	0.65	0.64	1.31	100	100
					10	1.10	0.55	0.60	1.26	100	100
Control	NA	NA	NA	NA	11	1.30	0.75	0.65	1.40	100	100
					12	1.25	0.75	0.55	1.27	100	100

The four modified reaches had a mean (± SD) wetted bank width of 5.82 m (± 2.73), a predominant flow type classed as ‘deep pool’ [29], a mean velocity (± SD) of 0.09 m s^-1^ (± 0.07), and depths that regularly exceeded 0.5 m (54%), with reach 3 exhibiting shallower areas comprising 85% < 0.5 m. In most areas the substrate was predominantly silt, except immediately below dams where gravel dominated [30]. For the control, the mean (± SD) wetted bank width was 0.8 m (± 0.26), the dominant flow type was classed as riffle, mean velocity was 0.27 m s^-1^ (SD ± 0.07), depths did not exceed 0.2 m (100% < 0.2 m), and the dominant substrate was pebble/cobble [30].

The fish fauna at the study site is dominated by freshwater-resident brown trout, accompanied by three spined-stickleback (*Gasterosteus aculeatus*) and European eel (*Anguilla anguilla*). In 2008 a breeding pair of Eurasian beaver of Bavarian origin were released into the loch situated within a 40-ha enclosure, incorporating ca. 1.2 km of available stream habitat and ca. 0.6 km of loch shoreline.

Five water level loggers (OTT Orpheus Mini, OTT Hydromet) installed in December 2014 (one above and below Dam 1 and 4 and one in the control stream, Fig 1) recorded water depth and temperature every 5 mins and averaged at 15 min intervals. The mean [± SD] river depth in the control stream during October and November was 0.10 m [0.08] in 2015 and 0.08 m [0.03] in 2016 (December was excluded due to logger error in 2016).

**Fig 2:**
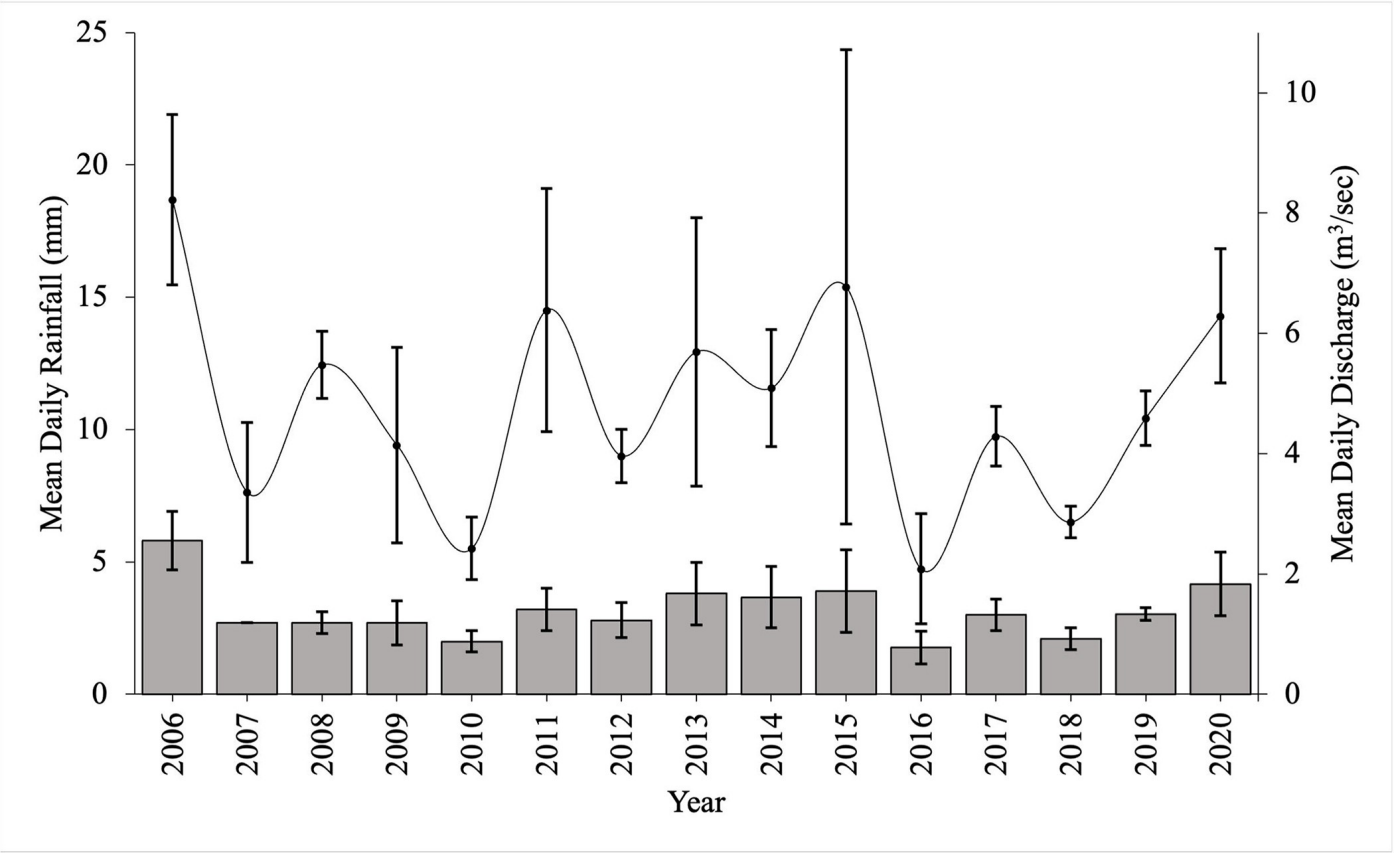
Coarse resolution rainfall and discharge data from a nearby river. Coarse resolution environmental data of mean [± SE] daily rainfall obtained from the Lentran Meteorological station and mean [± SE] daily river discharge for the river Enrick at Mill of Tore (NH4504429976, ~ 14km southwest of study site) for the Monitoring Period (October—December inclusive) from 2006–2020. Rainfall data is missing for October and November 2007 and December 2010 and 2017.

**Fig 3:**
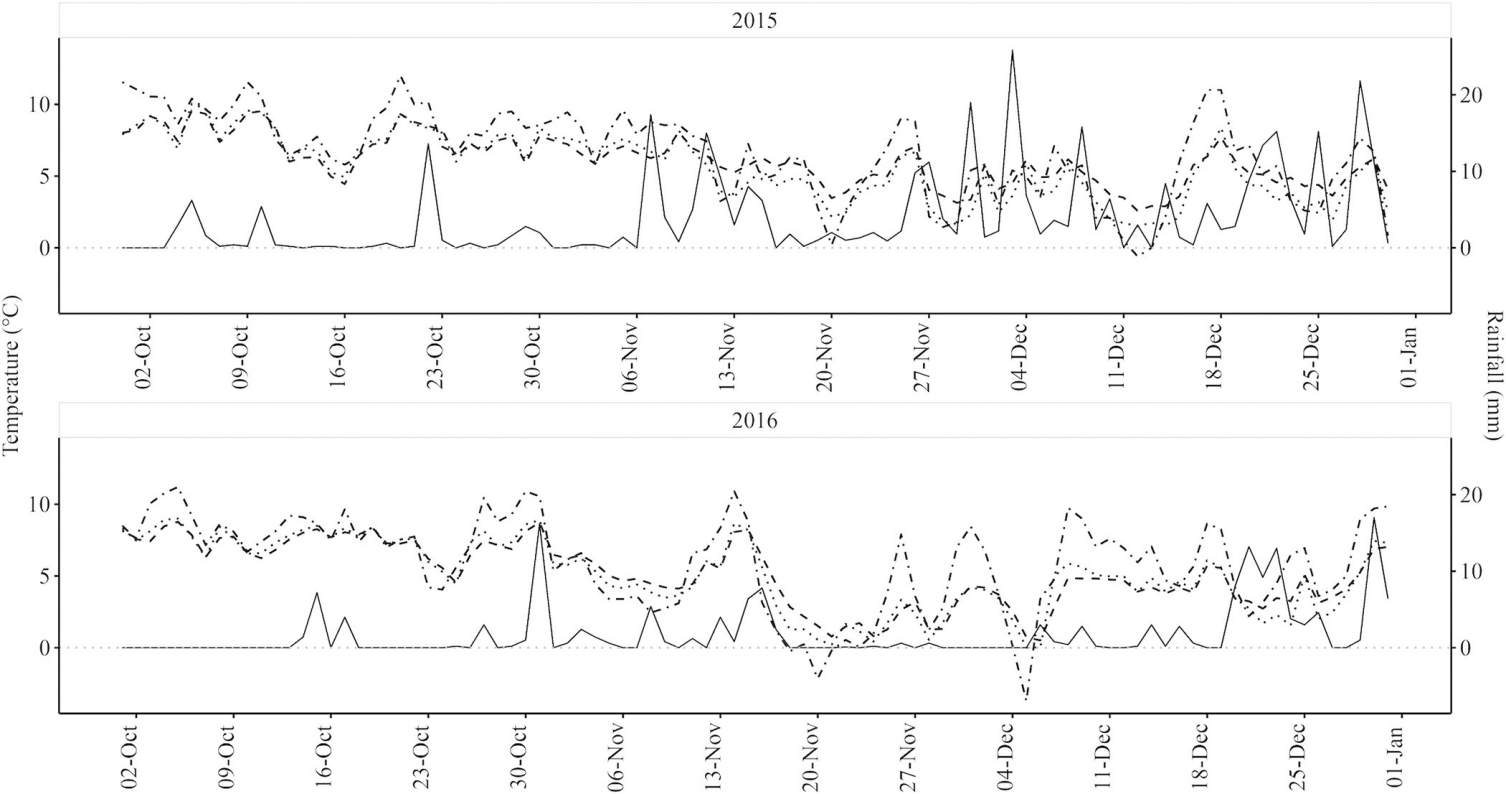
Environmental data for the monitoring periods in 2015 and 2016. Environmental data for the Allt Coire an t- Seilich (beaver modified) and Allt a’ Choilich (control) streams in Inverness-shire collected during the Monitoring Period (October–December inclusive), 2015 and 2016. The study investigated the influence of beaver dams on the spawning migration of brown trout. Solid, dot-dashed, dashed and dotted lines denote the daily means for rainfall, ambient air temperature, modified stream water temperature (based on an average of all four data loggers), and control stream water temperature, respectively.

In the absence of discharge data at the site our results were compared with a long-term river discharge data series obtained for the River Enrick at Mill of Tore (NH4504429976) ~ 14km southwest of the site between October and December from 2006–2020 to coincide with periods when brown trout spawn (Fig 2). Air temperature and rainfall data were obtained from the Met Office’s Lentran weather station situated ca 6 km northwest of the study site (NH577435; Fig 3 and Table 2).

**Table 2:** Summary of ambient air temperature, daily rainfall (mean [±SD]) and total rainfall for the monitoring period (October—December inclusive) 2015 and 2016. Data obtained from Met Office Lentran weather station situated ~ 6 km north-west of the study site.

	2015			2016		
Study Periods	Ambient Air Temp °C (Mean ± SD)	Daily Rainfall (mm) (Mean ± SD)	Total Rainfall (mm)	Ambient Air Temp °C (Mean ± SD)	Daily Rainfall (mm) (Mean ± SD)	Total Rainfall (mm)
October	8.95 (± 1.75)	1.32 (± 2.75)	41.0	8.29 (± 1.76)	1.07 (± 3.20)	33.2
November	6.07 (± 2.76)	3.66 (± 4.64)	109.8	3.77 (± 3.15)	1.18 (± 2.06)	35.3
December	4.80 (± 2.89)	6.75 (± 7.04)	209.2	5.74 (± 3.00)	3.01 (± 3.01)	93.2

The physical habitat characteristics of each reach were surveyed in May 2016, during spring baseflows following the Scottish Fisheries Coordination Centre methodology [29, 30]. In July 2016, wetted width and bathymetry of the modified and control streams were quantified using differential GPS (Leica Viva GS14 Smart Antenna and a Viva CS15 Controller) (Fig 1 and Table 3).

**Table 3:** Physical habitat characteristics of an unmodified (control) and beaver modified stream. Flow characteristic abbreviations are listed in order of dominance: SM—Still marginal; DP—Deep pool; SP—Shallow pool; DG—Deep glide; SG—Shallow glide; RU—Run; RI—Riffle and TO—Torrent. Flow velocity (0.001 m s ⁻^1^ resolution averaged over 60 s) 0.6 of the total depth, mid channel in the centre of the reach. Where flow types varied within reach one reading was taken in each flow type. Note. Mean wetted bank width in control reaches 1, 2 and 3 was greater than bank width due to heavily undercut banks.

Location	Length (m)	Mean wetted width (m)	Mean bank width (m)	Area (m^2^)	Depth (m)	Flow Type	Velocity readings (m^-s^) ± [SD]	Substrate Composition
Control Reach 1 *Riffle* *Pool*	40	0.52	0.45	20.75	<0.1	RI (100%)	0.23 ± 0.005-	Pebble (60%) Gravel (20%) Sand (20%)
Control Reach 2 *Riffle* *Pool*	26	0.65	0.53	16.87	<0.1	RI (100%)	0.38 ± 0.005-	Pebble (60%) Gravel (20%) Sand (20%)
Control Reach 3 *Riffle* *Pool*	42	1.00	0.96	42.17	≤0.2	RI (70%), SP (30%)	0.335 ± 0.033 0.132 ± 0.005	Pebble (30%) Cobble (30%) Gravel (20%) Sand (20%)
Control Reach 4 *Riffle* *Pool*	30	1.03	1.10	30.83	≤0.2	SP (70%), RI (30%)	0.372 ± 0.009 0.120 ± 0.007	Cobble (30%) Boulder (30%) Pebble (20%) Gravel (10%) Sand (10%)
Modified Reach 1 *Pool* *Pool*	50	4.20	4.95	215.53	>0.5	DP (90%), SP (10%)	0.022 ± 0.003 0.028 ± 0.004	Silt (90%) Gravel (5%) Pebble (5%)
Modified Reach 2 *Riffle* *Pool*	44	6.02	6.85	261.77	>0.5	DP (95%), RI (5%)	0.040 ± 0.003 0.041 ± 0.002	Silt (95%) Gravel (5%)
Dam 3 Bypass Channel *Riffle* *Pool*	30	0.78	0.87	37.60	≤0.2	SP (70%), RI (30%)	0.214 ± 0.019 0.204 ± 0.005	Silt (70%) Gravel (15%) Sand (15%)
Modified Reach 3 *Pool* *Pool* *Riffle* *Pool*	69	5.76	8.56	613.84	0.3 - >0.5	DP (80%), RI (15%), DG (5%)	0.048 ± 0.003 0.05 ± 0.003 1.101 ± 0.005 0.07 ± 0.002	Silt (65%) Gravel (20%) Sand (10%) Pebble (5%)
Modified Reach 4 *Riffle* *Pool* *Pool*	44	3.48	3.96	232.34	>0.5	DP (85%), DG (10%), RI (5%)	0.209 ± 0.006 0.071 ± 0.003 0.076 ± 0.005	Silt (65%) Gravel (25%) Sand (10%)

### Fish surveys

As part of another study [29] fish sampling and tagging commenced in 2014 prior to the start of the monitoring period (2015–2016) with fish detected being included in this study. Multiple capture methods were deployed to maximise sample size for telemetry, comprising electric fishing, netting and rod and line fishing. Electric fishing using a pulsed DC field generated by a control unit (Easyfisher EFU– 1, 2.5A maximum output, 50/100 Hz) was conducted in the loch, modified and control streams on six separate occasions (Table 4). Winged fyke nets (mesh size 2 cm) were set (at 20:00 and checked at 07:00) at the mouths of the control and modified streams to catch actively upstream moving trout during the nights of the 29–30 October 2014, and 27–29 October 2015. The nets were fitted with guards, a rigid square grille with bars separated by no more than 85 mm, to prevent accidental entry by Eurasian otter (*Lutra lutra*) and beaver. Rod and line fishing was used to increase sample size during the summer of 2016.

**Table 4:** Survey dates, locations, total *n* of PIT tagged fish (all PIT tag sizes and types), fork length (mm) (mean ± SD) and mass (g) (mean ± SD) of brown trout captured in the modified and control streams and loch between 2014 and 2016.

Year	Season	Location	Total n Tagged	FL (mm) [Mean ± SD]	Mass (g) [Mean ± SD]
2014	Autumn	Modified	88	147.02 ± 35.76	42.98 ± 44.03
		Control	0	0	0
		Loch	37	229.62 ± 46.415	144.16 ± 79.168
2015	Spring	Modified	76	96.84 ± 31.12	14.32 ± 17.17
		Control	6	69.50 ± 3.83	4.27 ± 0.83
		Loch	16	208.19 ± 92.88	147.67 ± 112.23
	Autumn	Modified	66	128.55 ± 35.03	28.22 ± 24.56
		Control	14	216.93 ± 70.60	134.36 ± 85.73
		Loch	48	249.52 ± 53.38	198.19 ± 107.08
2016	Spring	Modified	110	101.13 ± 25.06	14.50 ± 14.59
		Control	6	89.33 ± 10.82	10.60 ± 5.36
		Loch	0	0	0
	Summer	Modified	134	104.98 ± 24.75	16.14 ± 24.53
		Control	7	96.00 ± 8.35	11.57 ± 2.98
		Loch	26	205.65 ± 68.51	117.56 ± 103.47
	Autumn	Modified	39	110.46 ± 19.42	15.62 ± 7.71
		Control	1	88.00	8.1
		Loch	27	207.56 ± 65.28	116.09 ± 88.190
Total			701		

Between 2014 and 2016 a total of 701 individual trout were PIT tagged (Table 4). Captured fish were held in fresh aerated loch water for a maximum of 1 hour prior to being anaesthetized using 2-Phenoxyethanol (concentration; 0.2 ml l^-1^). Fork length (FL) and mass (g) were measured (Table 4), and individuals > 65 mm were tagged with half duplex (HDX) Passive Integrated Transponder (PIT); 65–180 mm FL = 12 mm HDX, n = 552, Oregon RFID, Portland, Oregon; > 180 mm FL = 23 mm HDX, n = 149, Oregon RFID, Portland, Oregon). Tags were inserted into the body cavity via a ventral incision and fish were allowed to recover for at least 1 hour prior to release. To assess the impact of tagging on survival and to quantify tag retention, a sample of trout (n = 16, FL = 192.8 ± 72.1 mm [mean ± SD] in 2014; n = 28, FL = 171.9 ± 97.1 mm [mean ± SD] in 2015; and n = 30, FL = 109.4 ± 19.7 mm [mean ± SD] in 2016) were retained post tagging and held for 48 hours in in-stream containers with through-flowing water. Tagged fish showed 100% tag retention and 0% mortality (n = 74). All fish were returned to the stream reach or loch from which they were originally captured.

### PIT telemetry

To establish movements of HDX tagged fish, half-duplex rectangular PIT loops (PLs) (2.5 mm^2^ cross-sectional area insulated wire consisting of 50 strands of 0.25 mm diameter copper wire) were constructed upstream and downstream of Dams 1, 2, 3 and 4 and two PLs were installed in the control stream in 2015 (Fig 1). This allowed direction of movement and successful passage to be determined. Due to the size of the dam and poor tag detection, the PL was removed and installed on the side stream of Dam 3 resulting thereafter in 100% tag detection efficiency (see below). Each PL was connected to a dynamic tuning unit (Wyre Micro Design, Model: DTU), PIT reader (Wyre Micro Design) and external data logger (Anticyclone Systems Ltd, Surrey, UK, Model: Antilog RS232) and powered by a 115 ah 12V leisure battery. PLs operated continuously from the 12 October to 12 December 2015 and from the 13 October to 31 December 2016. Detection range and efficiency of all PLs were established by passing both tag sizes (12mm and 23mm) through each PL at 30 random locations. Range and efficiency varied between 0.05–1.4 m and 97–100%, respectively (Table 1).

### Analysis

**Fig 4:**
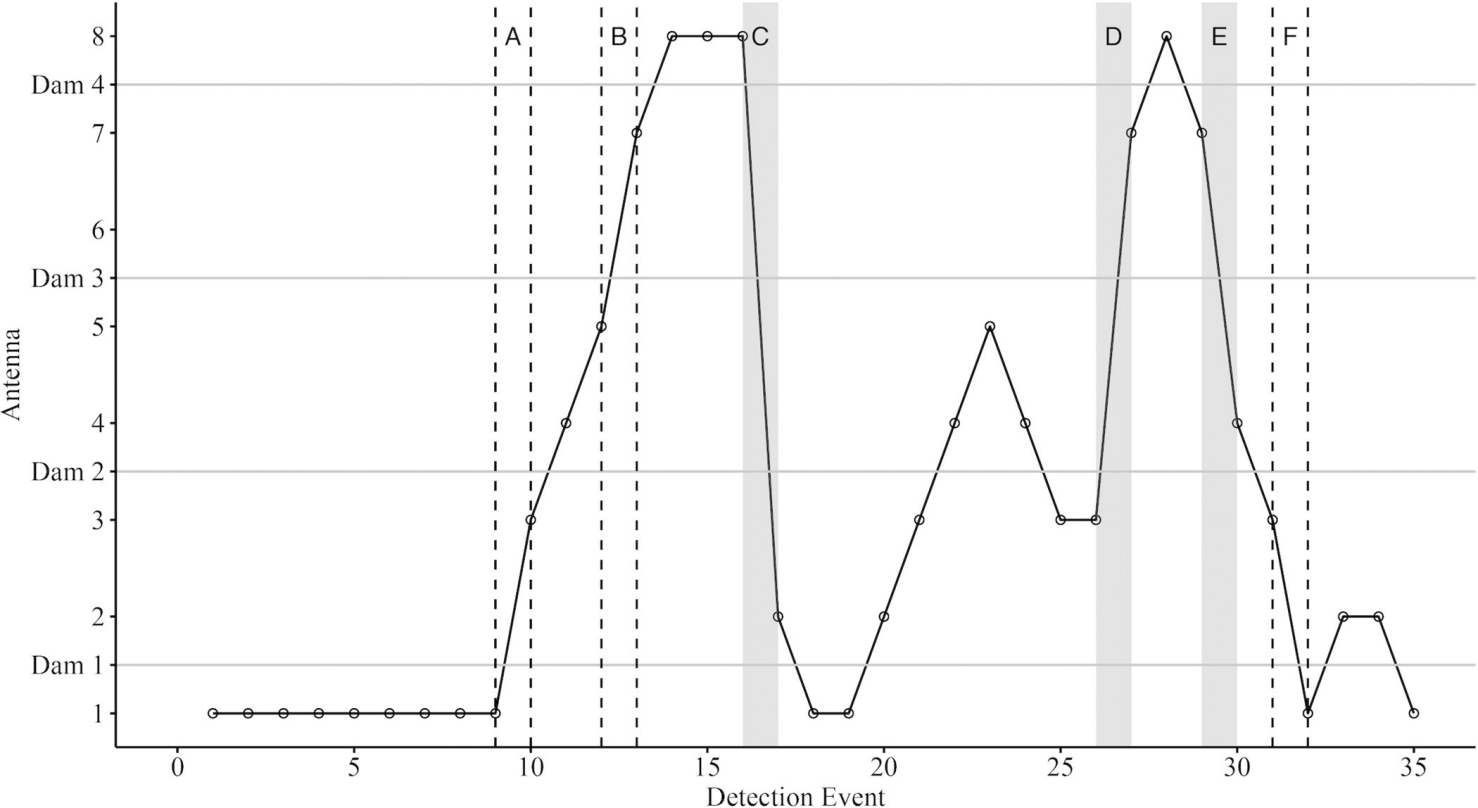
Movements of an individual PIT tagged trout. Movement patterns of an individual trout over an ~ca 10-hour period (between 18:22 on 25 October and 04:32 on 26 October 2016) exhibiting ‘abnormal’ movement patterns. Columns bordered by the dashed lines A, B and F represent ‘feasible’ movements in which the fish likely used the by-pass channels resulting in detection (circles on the solid black line) at antennas 1 and 3 (but missing antenna 2), and 5 and 7 (missing antenna 6 at Dam 3). Grey columns illustrate ‘abnormal’ behaviour when multiple dams appear to have been passed with the absence of detection at multiple antennas indicative of overland movements. The PIT tag of this individual was subsequently found on the bank of the modified stream using a handheld PIT antenna suggesting otter predation. Detection Event denotes each time the PIT tag was detected during the ~ca 10 hr period.

Prior to further analysis the quality of data of fish movements was assessed and abnormal behaviours identified. Cases where fish used bypass channels to move upstream (Fig 4A) were classed as successful passes. As only the bypass channel was covered by a PL (Antenna 6) at Dam 3, transition from antenna 5 to 7 was also classed as a successful pass (Fig 4B) because dam passage was feasible. In some cases, tags were detected at two isolated antennae without being recorded at the PLs in between (Fig 4C–4E). In 2015 these anomalies were rare. During the 2016 low flow year, however, such events were relatively frequent, and attributed to otter predation (a predator frequently capture on remote cameras installed at the study site) in which ingested PIT tags potentially bypassed the PLs when the otters moved over land, therefore these data were removed from further analysis due to the associated uncertainties.

### Passage efficiency

Upstream dam passage efficiency (PE) was calculated for all four dams in 2015 and 2016. PE was expressed as the number of fish that passed the dam, based on upstream detection, as a percentage of the total number of trout detected downstream (Table 4). As PLs were absent from the by-pass channel at Dam 1, successful passage via the bypass was deemed to have occurred if a fish detected below Dam 1 was subsequently detected below Dam 2. At Dam 3, where the downstream antenna covered the approach to both the dam and the by-pass channel, passage over the main dam structure was deemed to have occurred if a fish detected at PL5 was subsequently detected at PL7, immediately downstream of Dam 4. Individuals with only one recorded detection were removed from further analysis.

### Delay

Delay was quantified using survival (time-to-event) analysis to interrogate fish passage data, incorporating both rainfall (as a proxy for flow) and temperature (environmental factors) and fish size (biotic factor). Due to the limited data collected during the 2016 low flow year, data for both study years were aggregated. First, *T* was defined as a random variable, representing time to an event, with the event being dam passage. Next, *t*_*i*_ was defined as the observed passage time for the *i*^th^ individual. Individuals not observed to complete the event, either because they were lost or failed to pass by the end of the study, were designated as censored, with *c*_*i*_ representing their last observation time. These “right-censored” individuals contained important information that contributes to the estimation of passage rates [31] and were therefore included in the statistical analyses.

The survivorship function, *S(t)*, was the probability of the event not occurring before t days, or *S(t)* = *P(T > t)*. To visualize this function, the product limit, or Kaplan–Meier method [31, 32], was used by implementing the “survival” package in R [33], which estimates *S(t)* based on both observed and censored individuals.

Parametric models were used to analyse passage rates in relation to underlying model form and covariates, following the methods of Zabel et al. [34]. The fundamental feature of time-to-event modelling is the hazard function, *h(t)*, which is the conditional probability that the event will occur during the next short time increment, given that it has not yet occurred [35, 36]. A related function, the cumulative hazard function, *H(t)*, determined how much hazard an individual has experienced through time t, and is thus the integration of the hazard function through time t:

$$H(t)=\int_{0}^{t}h(t)d\tau$$ (1)

where *t* is a dummy variable for the integration. The survivorship function, *S(t)*, is the probability of the event not occurring before t days, or

S(t)=P(T>t)=exp(−H(t)) (2)

[31]. Note that the survivorship function was simply 1 − *F(t)*, where *F(t)* is the cumulative distribution function. Based on the hazard function, the probability density function of *t*, *f(t)*, is *f(t)* = *h(t)· S(t)*. Thus, once the hazard function is specified, all other functions necessary for statistical analyses are derivable from it.

To relate timing events to covariates, a standard assumption is that covariates acted multiplicatively on a baseline hazard function, *h*_*0*_*(t)*:

$$h(t)=h_{0}(t)exp\;(x^{\prime }\beta )$$ (3)

where **x** is a vector of covariates, and **b** is a vector of regression coefficients. This is equivalent to assuming that covariates act additively on the log hazard and has the desirable property that the hazard remains positive across all ranges of parameter values. This assumption forms the foundation of Cox Proportional Hazards modelling [37]. In this analysis, two parametric forms for the baseline hazard function, the exponential and Weibull distributions, were examined. The exponential model assumed that the baseline hazard is constant through time, or *h*_*0*_*(t)* = l. The Weibull model is more flexible where *h*_*0*_*(t)* is specified as al^a^*t*^a-1^. Note that if a = 1, the baseline hazard function reduces to the exponential function that will be used here as a null model. If a < 1, the hazard function decreases with time (survivorship function exhibits a type III response), and if a > 1, it increases with time (survivorship function exhibits a type I response). The likelihood function is expressed in terms of individuals observed to complete the event (at time *t*_*i*_) and censored individuals (last observed at time *c*_*i*_). For the censored individuals, it was known that their event time would have been > *c*_*i*_ if they were not censored. Accordingly, for censored individuals, *P(T > c*_*i*_*)* = *S(c*_*i*_*)* was included in the log-likelihood function:

$$L(\theta )=\prod_{i=1}^{N_{E}}f(t_{i}|\theta )\prod_{i=1}^{N_{C}}f(c_{i}|\theta )$$ (4)

where *N*_*E*_ is the total number of individuals known to complete the event, *N*_*C*_ is the total number of censored individuals, and **q** is a vector of model parameters that determine the hazard function. Substituting Eq (2) into the likelihood and taking the log, the log likelihood is:

$$\log\;L(\theta )=\sum_{i=1}^{N_{E}}\log\left[h(t_{i}|\theta )\right]-H(t_{i}|\theta )+\sum_{i=1}^{N_{c}}-H(c_{i}|\theta )$$ (5)

This formulation is flexible and can accommodate a broad range of hazard functions, including those applied in this analysis.

A suite of alternative models were developed where instantaneous fish passage rate was analogous to the hazard rate. All models were examined with either the exponential or Weibull model as the baseline model, *h*_*0*_*(t)*. Six covariates were analysed that were expected to affect fish passage rate: 1) temperature (°C); 2) rainfall during the initiation of fish passage (mm); 3) rainfall 24 hours prior to the initiation of fish passage (as a proxy for flow) (mm); 4) fish mass (g); 5) fish length (mm); and 6) an indicator function of whether the fish had previously passed. Each of the covariates was examined separately.

Model parameters were estimated by maximizing the log-likelihood function with respect to the parameters using the “optim” function in R and using the quasi-Newton optimization method [38]. The optim function was also used to numerically estimate the variance–covariance matrix. Akaike’s Information Criterion (AIC) was calculated for each alternative model, with differences between models (DAIC) > of approximately 2 or less providing support to the model with the lower value [39] and differences > 10 providing strong support. A model that only included the baseline hazard was treated as the null model, and models were compared with single covariates to the null model.

An AIC weight was also calculated for each model. AIC weight for the *i*th model is defined as

$$w_{i}=\frac{exp(-\frac{1}{2}\Delta_{i})}{\sum_{m=1}^{M}exp(-\frac{1}{2}\Delta_{m})}$$ (6)

where D_i_ is the delta AIC for the *i*th model in the candidate set and *M* is the total number of models in the candidate set. Values for *w*_*i*_ fall between 0 and 1, with values closer to 1 conferring more support for the model. *w*_*i*_ can be interpreted as the likelihood that a given model is the best among the candidate set [39].

To demonstrate prediction of event times for a specified set of conditions, passage times were predicted, and their uncertainty based on several levels of the covariates available for brown trout. For each covariate, passage distributions were predicted based on best-fit parameter estimates with the covariate set to 5th percentile, median, and 95th percentile values. To represent model uncertainty, parameter sets were randomly selected from a multivariate normal distribution with mean and variance respectively set to the parameter point estimates and the estimated variance–covariance matrix. In this manner, 1000 predicted passage distributions were generated based on specified covariate values. Based on the sample of predicted passage distributions, the upper and lower bounds of the middle 95% passage proportion were calculated for each hourly time step.

### Motivation

**Fig 5:**
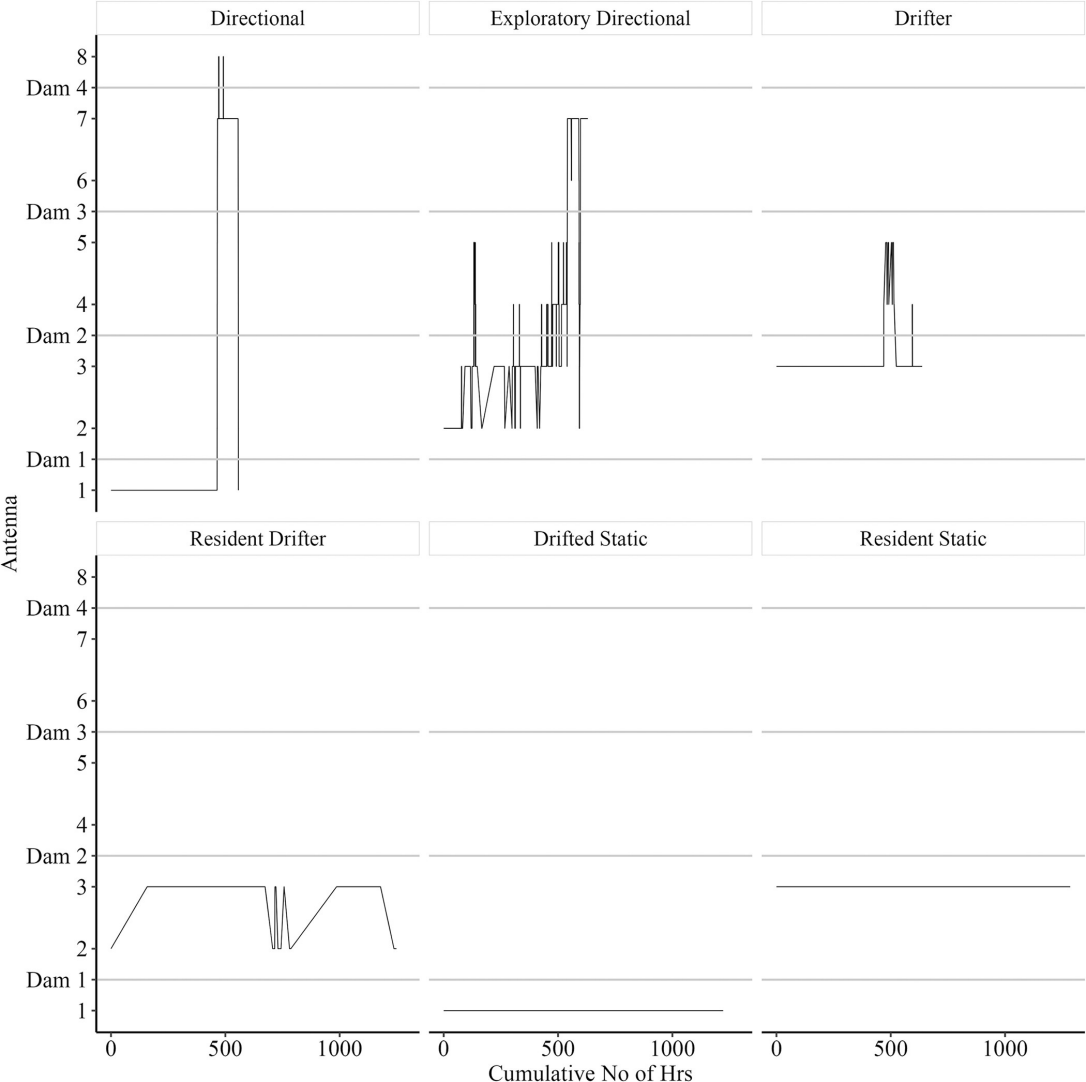
Movement patterns of six individual brown trout. Movement patterns of six individual brown trout during the 2015 Monitoring Period demonstrating the different behaviours observed for individuals in the modified stream and how they were grouped into one of six categories: (1) **Directional**, (2) **Exploratory Directional**, (3) **Drifter**, (4) **Resident Drifter**, (5) **Static** and (6) **Resident Static**. Location of beaver dams are illustrated in relation to antenna locations with zero representing the Loch.

To quantify motivation for individual trout during 2015, a two-stage approach was adopted. First, their movement as defined by detections at the dams were categorised into six generalised movement patterns (Table 5 and Fig 5), with 1 and 6 being the most and least motivated, respectively. The categories were defined as: (1) Directional—motivated directional movement; (2) Exploratory Directional—directional movement with searching behaviour in upstream and downstream directions; (3) Drifter—movement outside of the capture reach with no obvious direction or intent; (4) Resident Drifter—movement upstream and downstream within a single reach or loch outside of their original capture location; (5) Static—detected at a single PL outside of the original capture location; and (6) Resident Static—limited movement indicated by detection at a single PL within the capture reach.

**Table 5:** Brown trout movement patterns as defined by detections at dams and the relative number by tagging location.

Category	Definition	Total *n*	% Tagged in Loch	% Tagged in Modified Stream	% Tagged in Control Stream
Directional	Motivated directional movement	45	44.4	55.6	0
Exploratory Directional	Directional movement with searching behaviour in upstream and downstream directions	49	59.2	36.7	4.1
Drifter	Movement outside of the capture reach with no obvious direction or intent	17	0	52.9	47.1
Resident Drifter	Movement upstream and downstream within a single reach in which they remained after capture	11	0	100	0
Static	Detected at a single PL outside of the original capture location	13	69.2	30.8	0
Resident Static	Limited movement indicated by detection at a single PL within the capture reach	45	0	100	0

Second, swimming speed (m min^-1^) between the un-impounded sections of Dam 1–2, Dam 2–3 and Dam 3–4 was calculated for ‘Directional’, ‘Exploratory Directional’, ‘Drifter’ and ‘Resident Drifter’ categories, based on an assumption that more motivated individuals would swim more rapidly. Swimming speed between two dams was calculated as the last detection at the upstream antenna of the lower dam (e.g. Antenna 2 of Dam 1) and the first detection at the downstream antenna of the upper dam (e.g. Antenna 3 of Dam 2) (see Fig 1). In light of a further assumption that larger fish may be more motivated to move upstream (e.g. to spawn) than smaller fish, the relationship between fork length and swimming speed for representatives of the four most motivated categories was investigated.

Data was tested for assumptions of normality and homogeneity of variances using a Shapiro-Wilk’s normality test and Levene’s test, respectively. Outliers were assessed visually, and 3 extremes were removed. To investigate how swimming speed was influenced by movement pattern and location, a two-way ANOVA was used because it is considered robust to light deviations from the assumption of normality and homogeneity of variance [40] that were observed in this instance. Post hoc comparisons were performed using Tukey multiple comparisons of means. Differences in fork length between categories of movement pattern were tested using a Welch’s ANOVA as homogeneity of variance was strongly violated (p < 0.001). Post hoc comparisons were performed using the Games-Howell test. All statistical tests were conducted using R.

### Ethics

Field work was performed after review and approval by the University of Southampton’s Animal Welfare and Ethical Review Body (AWERB) following the 3R’s framework. Tagging was conducted in compliance with UK Home Office regulations under the Animals (Scientific Procedures) Act 1986, under project licence PPL 30/3196 and personal licence PIL ID71D59A5.

## Results

### Passage efficiency

During the 2015 Study Period, a total of 166 trout were detected at the PLs in the beaver modified stream. These comprised 93 tagged individuals captured in the loch, 71 from the modified stream and 2 from the control. Twenty-nine fish were only ever detected once having approached the upstream dam in the respective reach. For the remaining 137, the median number of successful passes was 3 (n = 142). The data was highly skewed with 12 (9%) making a single pass and 34 (25%) individuals responsible for multiple passage events (n = 130). A total of 53 trout were detected in the control stream, 32.1% (n = 17) exclusively so, with the remainder (67.9%, n = 36) also detected approaching Dam 1 in the beaver modified stream, indicating movement between streams via the loch (Table 6).

**Table 6:** Total number of trout detected at Dams 1–4 while moving in an upstream direction. Successful or failed passage attempts are recorded, enabling the calculation of passage efficiency. Routes taken are also depicted as are the % of resident individuals from within those reaches that did not pass.

	Dam 1		Dam2		Dam 3		Dam 4	
	2015	2016	2015	2016	2015	2016	2015	2016
Total Dam Passes (including repeat passers)	19	8	61	5	47	0	14	0
Detected Below Dam	59	39	38	22	50	21	72	26
Failed to Pass	40	33	8	17	14	21	58	26
Detected Upstream of Dam	6	0	30	5	NA	NA	15	0
Passed Side Channel	13	6	NA	NA	36	0	NA	NA
Total Passed	19	6	30	5	36	0	15	0
**Passage Efficiency (%)**	**32.2**	**15.4**	**79.0**	**22.7**	**72.0**	**0**	**21.8**	**0**
% Resident No Passage	NA	NA	100	76.5	84.6	85.7	43.9	76.00

In 2016, a total of 166 trout were detected at PLs in the beaver modified stream (including those entering from the loch). These comprised 30 tagged individuals captured in the loch, 132 from the modified stream and 4 from the control, with 66 detected only once having approached the upstream dam in the respective reach. For the remaining 100, the median number of successful passes was 1 (n = 13), with 6 (6%) making a single pass and 3 (3%) individuals responsible for multiple passage events. A total of 9 (9%) trout made 13 successful upstream passes. Twenty-three trout were detected in the control stream, of which 52.2% (n = 12) were exclusively so, while the remaining 47.8% (n = 11) also attempted to pass dams in the beaver modified stream. Upstream dam passage efficiency was higher in 2015 than in 2016 (Table 6). An equal number of fish were detected in the modified stream during both years, but a lower number were observed in 2016 (n = 23) than in 2015 (n = 53) in the control (Table 6).

### Delay

**Fig 6:**
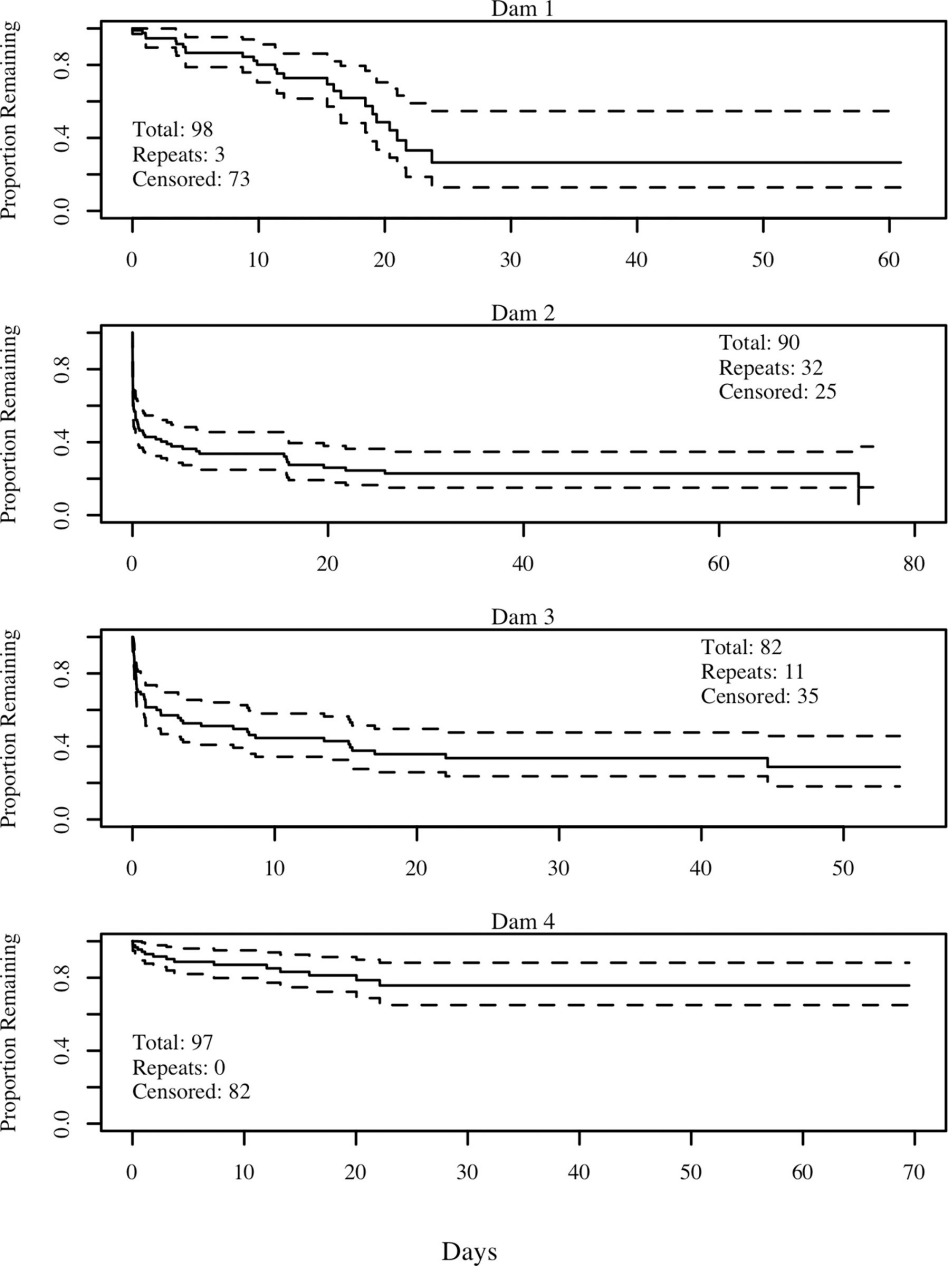
Kaplan Meier plots for all four beaver dams. Kaplan Meier plots for all four dams with data from 2015 and 2016 combined due to small dataset from 2016. The solid lines represent estimated proportion remaining in front of the dam, and the dashed lines represent 95% confidence intervals. In the legend, total refers to total number of fish tagged, repeats are the number of repeat passers, and censored is the number of fish that were never detected passing the dam.

Different patterns of passage were revealed at each dam (Fig 6), with relatively slow progress at Dam 1, where > 50% of the fish did not pass within 10 days. At Dams 2 and 3, passage occurred relatively quickly at first, with approximately 50% passage within the first day. At these dams, the remaining fish passed at a much slower rate, with the Survival distribution exhibiting a long tail. Passage was relatively slow at Dam 4, where only about 25% of the fish passed during the Study Period.

The parametric model analysis revealed interesting patterns. The Weibull model received strong support as a baseline hazard model for Dams 2–4, with the null models showing marked decreases in AIC values compared to the Exponential null models. In contrast, the Weibull model was not supported at Dam 1. For Dams 2–4, the α parameter was substantially lower than 1.0, indicating that hazard rate decreased with time and that the longer an individual was at the face of a dam, its instantaneous passage rate decreased. For Dam 1, the α parameter was not significantly different to 1.0 (based on its confidence interval overlapping with 1.0), adding further support to the exponential baseline hazard model.

**Fig 7:**
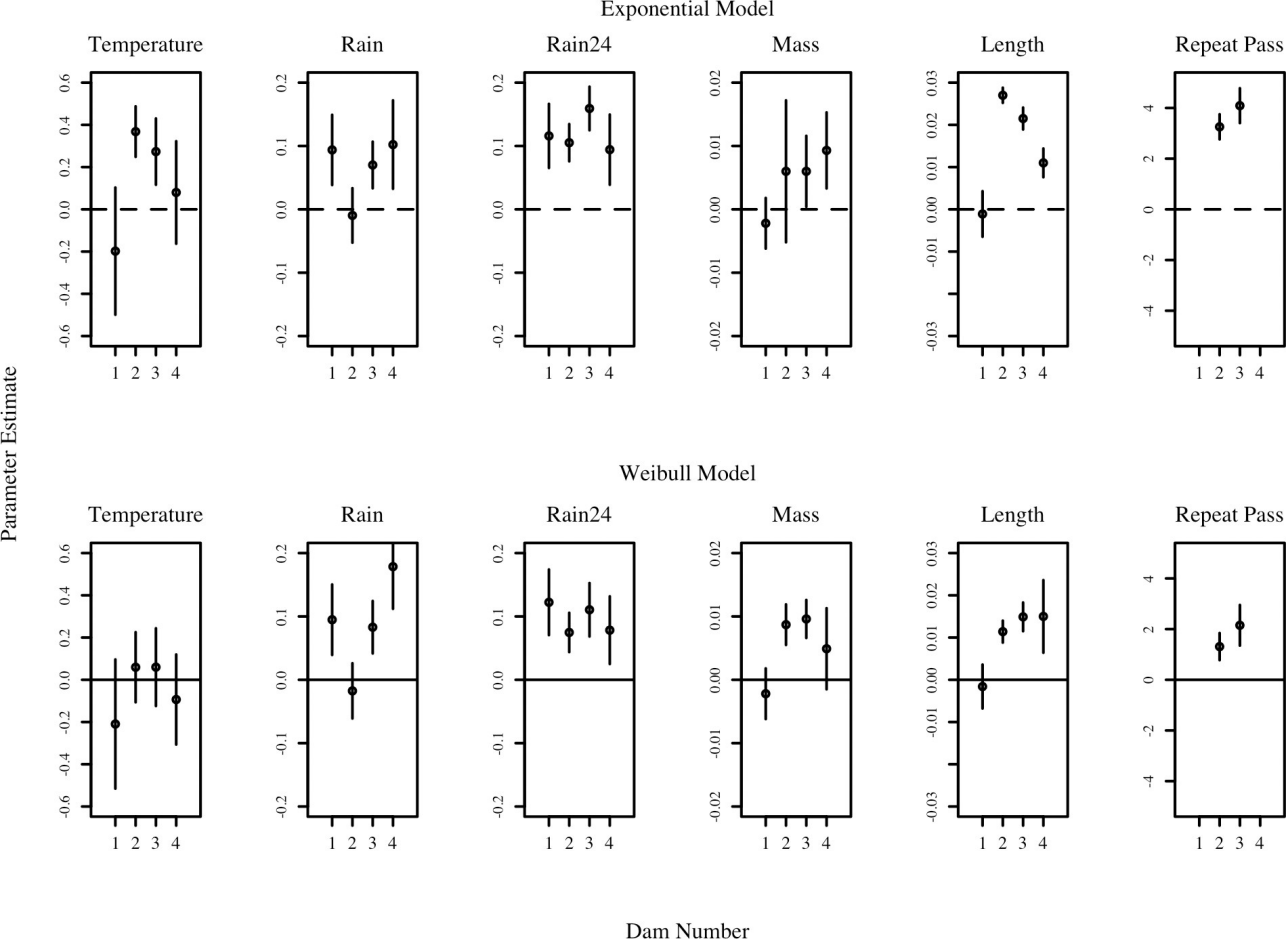
Covariate parameter estimates. Covariate parameter estimates (b coefficients) for water temperature, rainfall, rainfall with 24 hr lag applied, fish mass, fish length and repeat passers. Open points represent parameter estimates and vertical lines represent ± 2*(SE). If the vertical line does not intersect with zero, that provides evidence that the effect of the covariate is significant.

For the most part, the covariate estimates were similar regardless of the baseline hazard function. However, there were a few notable exceptions (Fig 7). Temperature was a significant covariate (based on its confidence interval not overlapping with 0) at Dams 2 and 3 with the Exponential, but not the Weibull, baseline model. In contrast, fish mass was a significant covariate at Dams 2 and 3 with the Weibull, but not so with the Exponential model.

Rainfall, as fish approached the dams, was typically significant (except at Dam 2), with a positive relationship with passage rate (Fig 7). Interestingly, Rainfall lagged by 24 hours was significant at all dams and had slightly greater magnitude. This implies that fish responded more to yesterday’s rainfall than todays, and thus the lagged rainfall provided a better proxy measure of discharge. Fish size, measured as both fish mass and fish length, was positively related to passage rate in most cases, particularly at Dam 2, 3 and 4 (Fig 7).

The AIC weights indicated that one model was overwhelmingly favoured for three out of four dams, although there was a different covariate that was deemed most important for each: Dam 1 = rainfall lagged 24 hours; Dam 2 = repeat passage; and Dam 3 = fish length. For Dam 4, rainfall lagged 24 hours and fish length both received strong weights. Interestingly, the weighting was similar regardless of which base model was used.

**Fig 8:**
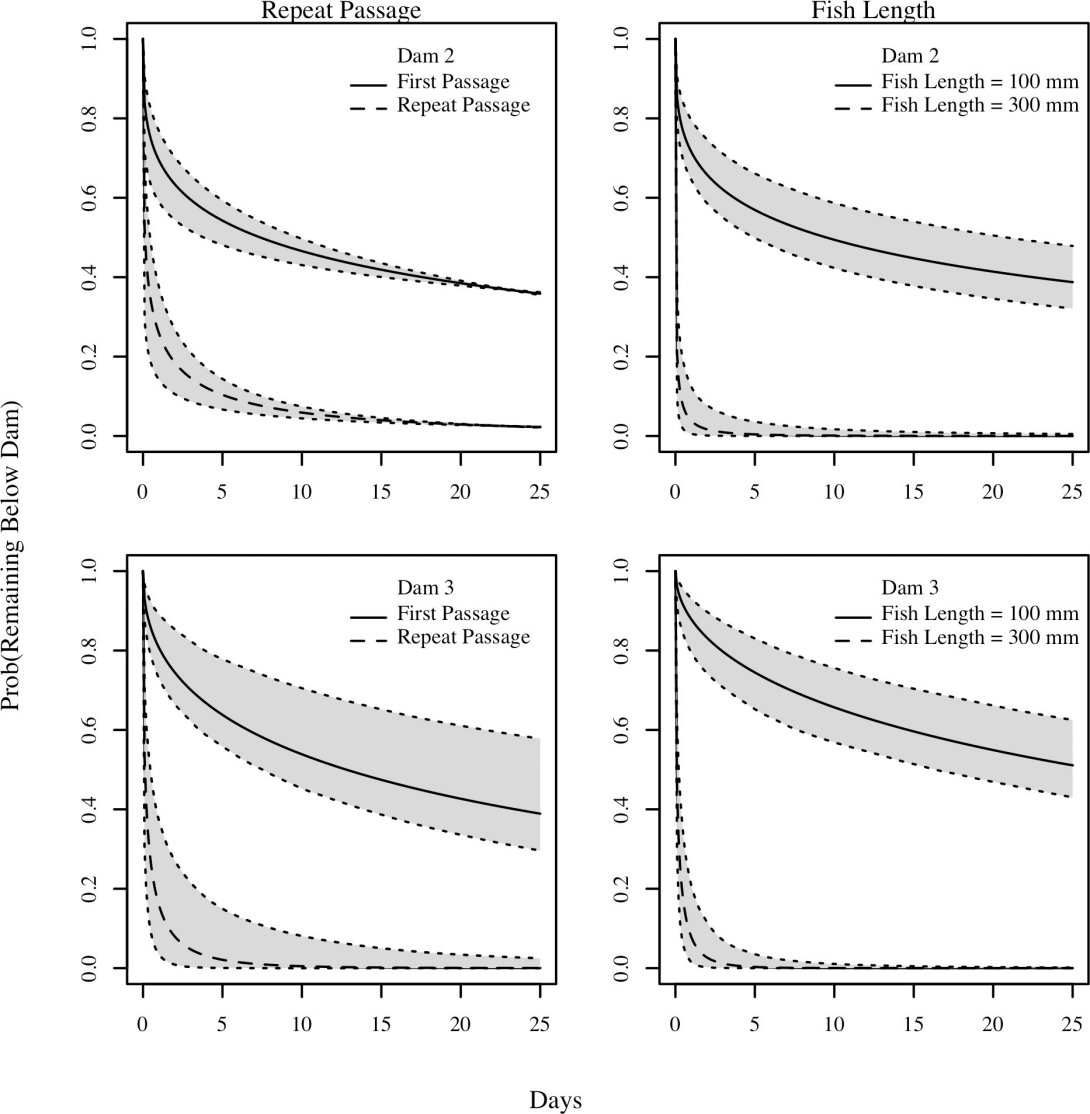
Passage prediction plots. Passage prediction plots for: (1) fish that passed a dam for their first time and for fish that had already passed a dam, and (2) fish with fork length of 100 mm vs fish with fork lengths of 300 mm. The solid and dashed lines represent estimated proportion remaining, and the grey areas represent 95% prediction intervals.

The repeat passers exhibited interesting dynamics (Fig 7). Fish that had previously passed a dam passed it at a much quicker rate the second time around. The confidence intervals about these covariates were relatively tight. The strong increase in passage rate is demonstrated by using the model predictively (Fig 8), with multiple passers passing Dams 2 and 3 much more quickly once they had already passed the dam previously. When using the model predictively for fish length the results demonstrate that fish with a fork length of 300 mm pass Dams 2 and 3 much faster than fish with a fork length of 100 mm (Fig 8).

### Motivation

**Fig 9:**
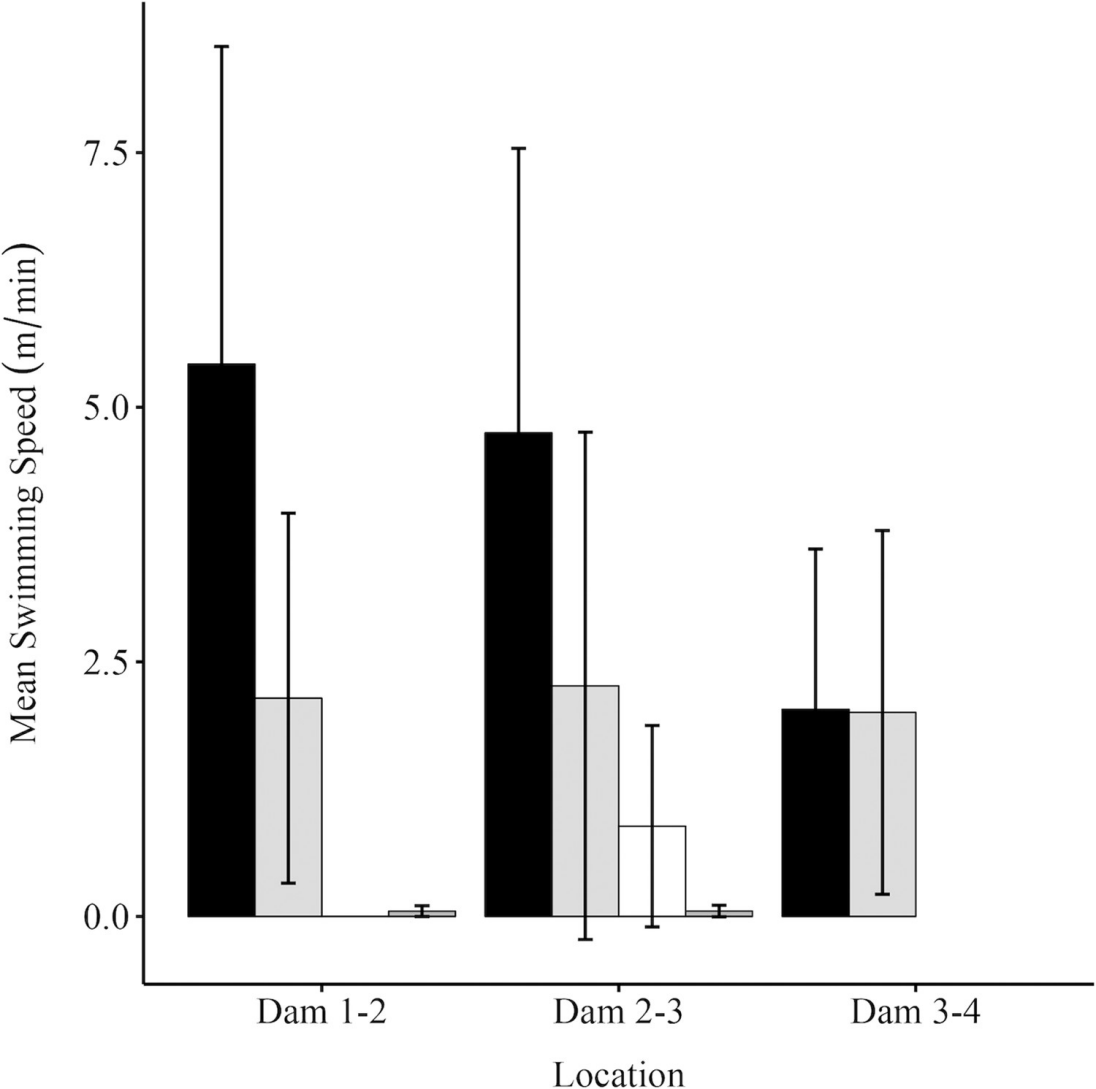
Mean swimming speed (m min^-1^) between beaver dams. Mean swimming speed (m min^-1^) between Dams 1–2, 2–3 and 3–4 for each movement category including ‘Directional’ [black bars], ‘Exploratory Directional’ [light grey bars], ‘Drifter’ [white bars] and ‘Resident Drifter’ [dark grey bars]. Error bars denote standard deviation.

Fish representing the different categories of movement pattern exhibited different swimming speeds between the dams (F _(3, 77)_ = 8.372, p < 0.001), which also varied with location (F _(2, 77)_ = 3.868, p = 0.025) (Fig 9). There was no interaction effect. Swimming speed differed between all groups, with the exception that ‘Drifter’ (0.709 [SD = 0.94] m/min) was not significantly different to ‘Exploratory Directional’ (2.12 [SD = 2.05] m/min) or ‘Resident Drifter’ (0.05 [SD = 0.05] m/min). The fastest swimming speeds were exhibited by representatives of the ‘Directional’ (3.61 [SD = 2.71] m/min) category, whereas the ‘Resident Drifters’ were the slowest (0.05 [SD = 0.05] m/min). Swimming speed differed between dams, with fastest between Dams 2–3 (2.69 [SD = 2.83] m/min) and slowest between Dams 3–4 (2.02 [SD = 1.67] m/min).

**Fig 10:**
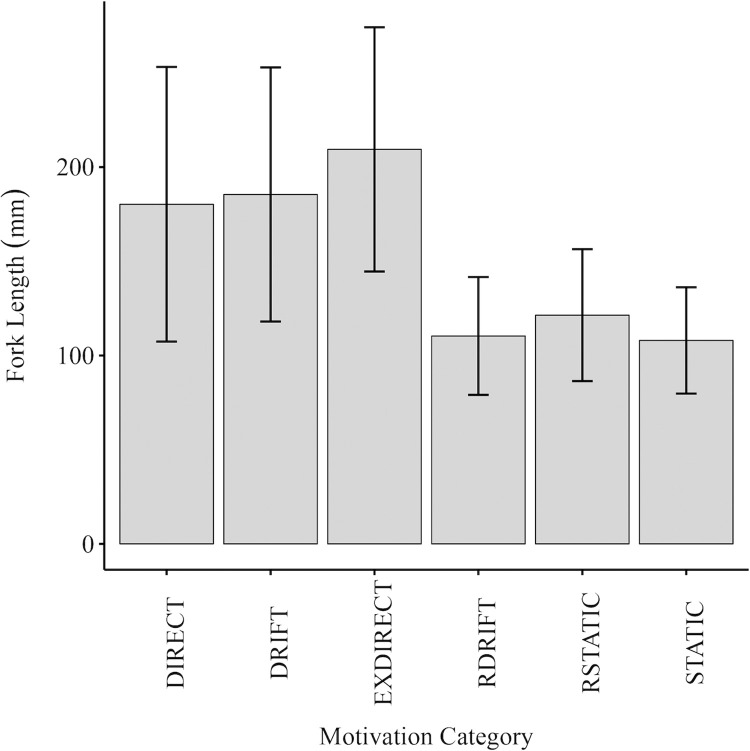
Mean [± SD] fork length of trout for each movement category. Mean [± SD] fork length of trout captured from the Loch, Modified stream and the control stream, for each movement category. Error bars denote standard deviation.

The different categories of movement pattern (F_(6, 27.52)_ = 18.092, p < 0.001) were represented by fish of differing mean size (FL) with those assigned to the Directional (180 [SD = 72.9] mm), Exploratory Directional (209 [SD = 64.8] mm), Drifter (185 [SD = 67.5] mm) categories tending to be the largest, while those belonging to the Resident Drifter (110 [SD = 31.3] mm), Static (108 [SD = 28.2] mm) and Resident Static (121 [SD = 35.0] mm) tending to be the smallest (Fig 10).

## Discussion

This study investigated the impact of Eurasian beaver dams on the upstream movements of brown trout in Northern Scotland, UK. The study was conducted over two years, with high rainfall (24% higher than the 14-year mean, 2006–2020) and river flows (42% higher recorded at a gauging station on a nearby river) experienced during the Monitoring Period (autumn and early winter) in 2015. Conversely, in 2016 rainfall and flow was substantially lower (44% and 69%, respectively) than the 14-year mean. The 2016 low flow year likely represents conditions that are predicted to prevail more frequently in the future, during periods that include salmonid spawning movements, due to climate change [26, 27]. Beaver dams in the modified stream created four potential barriers to the upstream movements of brown trout. As predicted (P1) passage efficiency was much higher in 2015 than 2016, in association with the greater rainfall recorded during the spawning period; rainfall with a 24-hr lag applied was a significant positive covariate in passage rate, providing a strong proxy for river flow. In both years, passage efficiency varied between the dams, with Dams 1 and 4 recording lower passage efficiencies in 2015. During the low flows and periods of below freezing temperature of 2016, the dams represented a much more significant impediment to movement than in the previous year, with lower passage rates (P1) and greater delay (P2) recorded at Dams 1 and 2, while Dams 3 and 4 represented a complete barrier (i.e., zero passage recorded). Survival analysis revealed that delay varied among dams, with passage being relatively slow at Dams 1 and 4, but rapid at Dams 2 and 3, at least at first with approximately 50% of fish passing within the first day. Passage at the dams was bidirectional, with a number of individuals falling back and making multiple repeat passes, with fish that had already passed passing much quicker when they attempted a second time. There was also considerable variation in movement behaviour at the dams, with those trout demonstrating more motivated patterns tending to swim most rapidly between them. Larger fish also tended to be more motivated, with fish size a significant positive predictor of passage rate at most dams, and larger fish typically passing the dams more quickly than smaller individuals (P3). Water temperature also proved to be a significant positive predictor in passage rate at Dams 2 and 3, but not Dams 1 and 4.

The potential for beaver dams to impede the movement of fish, and commercially important salmonids in particular, is an area of concern in relation to their reintroduction [14, 19]. Passage efficiency varies with both extrinsic environmental variables (e.g., dam characteristics, flow/ rainfall, temperature) and endogenous biotic factors (e.g., motivation and fish size). As observed for other salmonids in North America, such as Bonneville cutthroat trout (*Oncorhynchus clarkii utah*) [11], this study demonstrated that brown trout are quite capable of passing beaver dams, particularly during periods of moderate flow. Unlike in the case of human engineered dams, if rivers are allowed to respond naturally to the construction of beaver dams, they tend to be rather “leaky” to the movement of fish. Out of the four dams considered in this study, two had associated bypass channels that fish used to navigate as the preferred route of passage (68% at Dam 1; 100% at Dam 3 in 2015). Of particular interest is that during a period of unusually low flow during the 2016 Study Period, the ability of trout to pass the dams was significantly impeded, with zero passage occurring at some, and the presence of a bypass channel at one (Dam 1) being the only successful route of passage. Whilst we were unable to monitor flow directly at out our study site, rainfall with a 24-hr lag applied provided a viable proxy with which passage was strongly correlated. The interaction between flows and beaver dams have been commented on by others, including in relation to impeded migration of Atlantic salmon in Catamaran Brook, New Brunswick, Canada [13] and Brierly Brook, Nova Scotia, Canada [12], and brown trout in Utah, United States of America [11]. Although, such negative impacts of beaver dams on fish movements are likely to be relatively temporary, the predicted increase in frequency and intensity of drought during the summer and Autumn months [25–27] requires further consideration from the perspective of fisheries management.

In addition to flow, temperature is also a critical determinant of migratory success in fish [41]. Physiological performance tends to be optimized over a relatively narrow range of environmental temperatures [42], which for salmonids tends to be low as they are cold-water adapted [43]. As swimming performance in salmonids is positively related to water temperature [44], exposure to low water temperatures will result in reduced ability to pass barriers. In this study, in contrast to 2015, low flows also coincided with low temperatures during the 2016 Study Period in which temperatures fell to below 0°C on several occasions and ice covered the loch and beaver ponds for prolonged spells. As a result, dam passage and entry into the control stream, which experienced a 56% reduction in the total number of individuals detected between the two years, was hindered. Reduced passage at natural barriers, such as waterfalls, due to low temperatures have been recorded for other salmonids, including Atlantic salmon [45]. The influence of temperature on passage rate is likely to interact with fish size, as both factors influence swimming performance [44]. Indeed, in this study larger fish were more likely to pass the dams as expected, although unfortunately comparisons between years were not possible due to the limited passage data available for 2016. Interestingly, larger fish are likely better able to tolerate reduction in temperature, particularly if the fluctuations are relatively small and rapid as has been seen in brown trout [46]. Further research is needed to determine whether the movements of smaller trout are more likely to be adversely impacted by beaver dams at low temperature, and how fish size, temperature and flow interact to influence passage.

Barriers, whether anthropogenic or natural, are known to delay the movement of fish [47]. Migratory delay can have substantial ecological consequences in relation to elevated energetic expense e.g. Atlantic salmon at small weirs [48], increased risk of predation for Pacific salmonids at large dams [49] and altered timing of arrival at the final destination, e.g. spawning site and Atlantic salmon [47]. Delay can vary widely with barrier, including those that would appear to have similar characteristics [47], an observation supported by the results of this study in which passage at some dams was relatively rapid, while slow at others, and varied with time (e.g. most rapid passage occurred within the first day). Rate of passage varied among individuals, indicating heterogeneity in passage behaviour, as illustrated by the results of the survival analysis. This approach provides mechanistic insights to fish passage at barriers by accommodating the number of fish that passed and tried to pass but failed, the number of attempts required, and the effort expended by fish that were ultimately successful as well as those that failed, and the duration of effort and availability of fish to pass or fail [50]. In our study, we tested the ability of two models to capture the passage dynamics of trout. The more complex Weibull base model generally received more support than the exponential base model, possibly being better able to accommodate the variability in passage behaviour among individuals, such as rapid passage by some followed by slower passage by the residual population, as indicated by the longer tail in the passage distribution.

Intraspecific variability in fish passage performance, and for brown trout in particular, is expected considering well recognised variation in migratory strategies [51, 52], swimming performance [53] and movement behaviours [21]. It is assumed that motivation to move and negotiate barriers, likely driven by internal physiological state [41, 51], will play an important role in the observed variation in fish movement patterns, but to date this has received limited consideration in the realms of fish passage research [21]. In this study, trout exhibited a range of movement behaviours that we categorised into six separate patterns and included bidirectional movements and repeated attempts to pass the dams. We defined a hierarchy of motivation based on the movement behaviours exhibited, with those demonstrating directed upstream swimming considered to be the most motivated, while those that remained resident in the area in which they were initially collected the least. More motivated fish, exhibiting “Directional” and “Exploratory Directional” movements were more likely to successfully pass the dams and moved more rapidly between them. These fish also tended to be larger, and presumably more likely to be in spawning condition, compared to those that were more stationary. Clearly, motivational status and movement patterns should be considered when estimating passage efficiency with those participating in directed movements towards an end goal (e.g., spawning grounds) accommodated.

This study has important implications for river management, particularly considering the challenges of a shifting climate in which interactions between flow, temperature and biotic factors are likely to influence the ability of fish to negotiate beaver dams. Consideration of such dynamics are nuanced when acknowledging the multiple positive benefits beaver modification can have for fish populations and wider ecological status more generally [14, 15, 53, 54]. This is particularly so when less managed natural river systems are allowed to respond more naturally, in which case beaver dams are less likely to present severe barriers to fish movements. However, in cases where rivers are further removed from their reference condition and lateral connectivity has been degraded, e.g. due to channelisation and incision, or in areas containing sensitive salmon populations, care is required to manage the systems appropriately.
